# Supplementary material for: High-brightness scalable continuous-wave single-mode photonic-crystal laser
Source: Nature. 2023 Jun 14;618(7966):727–32. doi: 10.1038/s41586-023-06059-8 (PMC10284696; doi:10.1038/s41586-023-06059-8)
Supplement: Supplementary file 1 — Supplementary Information Supplementary Figs. 1–3, Table 1 and Text. [file 41586_2023_6059_MOESM1_ESM.pdf]

nature portfolio

https://doi.org/10.1038/s41586-023-06059-8

Supplementary information

High-brightness scalable continuous-wave single-mode photonic-crystal laser

In the format provided by the authors and unedited

SUPPLEMENTARY INFORMATION

High-brightness scalable continuous-wave single-mode photonic-crystal laser

Masahiro Yoshida[1,3], Shumpei Katsuno[1,3], Takuya Inoue[2,3], John Gelleta[1], Koki Izumi[1], Menaka De Zoysa[2], Kenji Ishizaki[2], and Susumu Noda[1,2,*]

[1] Department of Electronic Science and Engineering, Kyoto University,

[2] Photonics and Electronics Science and Engineering Center, Kyoto University,

Kyoto 615-8510, Japan

[3]These authors contributed equally to this work

[*]E-mail: snoda@kuee.kyoto-u.ac.jp

1. Typical CW performance of conventional semiconductor lasers

As mentioned in the main text, conventional semiconductor lasers have limitations regarding their single-mode output power under CW operation. For example, in a conventional edge-emitting-type semiconductor laser, the maximum stripe width that supports oscillation in a single lateral mode is limited to approximately 10 μm, and the CW output power is limited to around one watt [S1]. In addition, we should note that these lasers also oscillate in many longitudinal modes.

Toward expanding the stripe width, the adoption of tapered stripes has been investigated. However, such stripes significantly worsen the asymmetry and astigmatism of the beam, resulting in a beam profile that is much different from an ideal Gaussian one [S2]. Similarly, a conventional vertical-cavity surface-emitting laser (VCSEL) also suffers from oscillation in many modes when its emission diameter is widened beyond approximately 5 μm, and its single-mode CW output power is limited to a few milliwatts [S3].

2. Comparison between PCSELs based on Hermitian/non-Hermitian control and those based on an open-Dirac singularity

In the present work, we report on large-scale CW single-mode PCSELs based on Hermitian/non-Hermitian control. As a different approach toward realizing scalable single-mode surface-emitting lasers, utilization of an open-Dirac singularity in a photonic crystal has been recently proposed [S4]. In the following, these two approaches are briefly compared.

The lasers based on an open-Dirac singularity, which can be also categorized as PCSELs, feature triangular-lattice photonic-crystal structures with symmetric circular

lattice points which are carefully tuned to realize the open-Dirac singularity. Since this structure possesses C_{6v} symmetry, the vertical radiation constant α_v of the lasing mode converges to zero when the resonator size is widened to larger scales. In addition to α_v, in actual semiconductor lasers, a fixed amount of fundamental material absorption loss α_0 (typically ≥ 1 cm^{-1}) exists due to free-carrier absorption in the cladding layers, etc. Considering these facts, the slope efficiency (i.e., surface emission efficiency) of the laser, which is proportional to $\alpha_v/(\alpha_v+\alpha_{//}+\alpha_0)$ where $\alpha_{//}$ denotes the in-plane loss, inevitably converges to zero at larger scales. The random scattering of light induced by fabrication disorders may recover the slope efficiency to some extent [S5] while sacrificing the beam quality. In addition, owing to the C_{6v} symmetry, α_v of modes on different band edges converge toward zero as the device size increases, so competition between the modes of different band edges might hinder single-mode operation. It should be also noted that, in [S4], rigorous simulations of *I-L* characteristics considering carrier-photon interactions were not performed, and experimental demonstrations were limited to resonator (emission) sizes of 64 μm under pulsed optical pumping (not under CW current injection).

On the other hand, in our approach based on Hermitian/non-Hermitian control in a double-lattice PCSEL, which has no rotational symmetry, the radiation constant of the lasing band-edge mode (mode A) can be controlled to an appropriate value while keeping those of the other band-edge modes (modes B, C, and D) much higher [S6], even at large scales. In this way, lasing in a single mode can be obtained while also ensuring a high slope efficiency and a high-beam quality single-lobed beam pattern. A detailed comparison between the approach based on an open-Dirac singularity and the approach based on Hermitian/non-Hermitian control is summarized in Table S1 below.

Table S1| Comparison between PCSELs based on an open-Dirac singularity and those based on Hermitian/non-Hermitian control

	PCSELs based on open-Dirac singularity ([S4])	PCSELs based on non-Hermitian/Hermitian control (this work and [S6])
**Structure**		
Periodicity	Triangular-lattice	Square-lattice (Double-lattice)
Unit cell and symmetry	Single circular hole (C_{6v} symmetry)	Elliptic and circular holes (Mirror symmetry along y=x, but no rotational symmetry)
Cladding	Air	Semiconductor
**Basic properties**		
Radiation constant of the lasing band-edge mode	Converges to zero for large devices (C_{6v} symmetry)	Arbitrarily tunable (no rotational symmetry)
Slope efficiency (considering material absorption loss)	Converges to zero when the size increases and disorders do not exist.	Can be kept high
Beam shape	Multi-lobe for small size, and random beam for large size	Single-lobed
**Simulation**		
Methods	3D finite-element method	3D coupled-wave theory
Device size	Up to 130 μm	3-10 mm
Carrier effect	Not considered	Considered
Lasing spectra	Not shown	Single mode
I-L characteristics	Not shown	50-100 W for 3 mm<br>500-1000 W for 10 mm
**Experiment**		
Device size	Up to 65 μm	3 mm
Excitation	Optical pumping	Electrical injection
Operation	Pulsed	CW
Output power	Not shown	CW 50W
Beam shape	Multi-lobed for small size (Up to 65 μm)	Single-lobed
Brightness	Not shown	1 $GWcm^{-2}sr^{-1}$ (rivals bulky lasers)

3. On the Hermitian coupling coefficients *R* and *I*

In this section, we explain the Hermitian coupling coefficients *R* and *I*, which are defined as $R \equiv \mathrm{Re}[(\kappa_{1\mathrm{D}} + \kappa_{2\mathrm{D}-})e^{-i\theta_{\mathrm{pc}}}]$ and $I \equiv \mathrm{Im}[(\kappa_{1\mathrm{D}} + \kappa_{2\mathrm{D}-})e^{-i\theta_{\mathrm{pc}}}]$.

Figure S1 shows the schematic illustration of modes A and C, which are expressed with a pair of electric-field vectors R_x+R_y and S_x+S_y. These two combined electric-field vectors are coupled with each other by $\kappa_{1\mathrm{D}} + \kappa_{2\mathrm{D}-}$ and its complex conjugate (see also Eq. (5) in the Methods section). Here, we should note that there is a degree of freedom in determining the phase of $\kappa_{1\mathrm{D}} + \kappa_{2\mathrm{D}-}$, which depends on the position of the air holes relative to the boundary of the unit cell. To determine the phase of $\kappa_{1\mathrm{D}} + \kappa_{2\mathrm{D}-}$, we use the position at which non-Hermitian coupling occurs as the reference position [S6]. We thus define the Hermitian coupling coefficient as $(\kappa_{1\mathrm{D}} + \kappa_{2\mathrm{D}-})e^{-i\theta_{\mathrm{pc}}}$, by multiplying $e^{-i\theta_{\mathrm{pc}}}$ to $(\kappa_{1\mathrm{D}} + \kappa_{2\mathrm{D}-})$. *R* and *I* are then defined as $R \equiv \mathrm{Re}[(\kappa_{1\mathrm{D}} + \kappa_{2\mathrm{D}-})e^{-i\theta_{\mathrm{pc}}}]$ and $I \equiv \mathrm{Im}[(\kappa_{1\mathrm{D}} + \kappa_{2\mathrm{D}-})e^{-i\theta_{\mathrm{pc}}}]$, respectively, as described above.

The real part *R* expresses the overall strength of in-plane feedback of combined

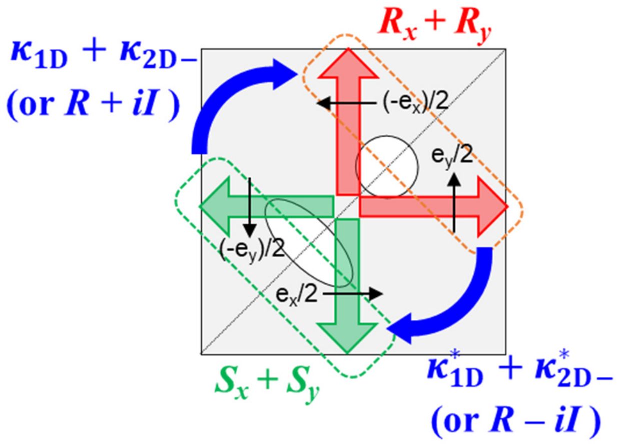

Fig. S1| Hermitian coupling coefficients for modes A and C. Modes A and C are expressed with pair of electric field vectors R_x+R_y and S_x+S_y with unit vectors of $\mathbf{e}_x$ and $\mathbf{e}_y$ in the *x* and *y* directions. R_x+R_y and S_x+S_y are coupled with each other by $\kappa_{1\mathrm{D}} + \kappa_{2\mathrm{D}-}$ and its complex conjugate.

180° and 90° diffractions, which determines the size of the frequency gap between modes A and C. Meanwhile, the imaginary part I determines the phase of the in-plane electric fields, namely, the position of the electric-field node with respect to the position of the air holes, and consequently determines the degree of cancellation of the vertical radiation in mode A at the Γ point: When I is zero, the vertical radiation is completely cancelled out, and when I becomes non-zero, the light begins to leak out in the vertical direction at a strength proportional to the non-Hermitian coupling coefficient μ. It should be noted that the imaginary part I is always zero in the case of a single-circular-hole photonic crystal that has C_2 rotational symmetry, and that the magnitude of I can be arbitrarily controlled via adjustment of the asymmetry of the two holes in a double-lattice photonic crystal as described in the next section.

4. Control of *R*, *I* and μ in a double-lattice PCSEL with a backside DBR

We explain here how the Hermitian coupling coefficients (R and I) and the non-Hermitian coupling coefficient (μ) can be controlled in a PCSEL (see Fig. S2a) with a double-lattice photonic crystal and a backside DBR mirror. As shown in the inset of Fig. S2a, the double-lattice photonic crystal is composed of elliptical and circular air holes, where the two air holes are separated by d and the size balance of the two air holes is expressed with the parameter of x; specifically, when the size of one of the air holes is increased by x, the size of the other air hole is reduced by x. The calculated real and imaginary parts of $\kappa_{1\mathrm{D}} + \kappa_{2\mathrm{D}-}$ as functions of d and x are plotted in Fig. S2b, where the axes of R and I are rotated by -θ_{pc} with respect to the axes of the real and imaginary parts of $\kappa_{1\mathrm{D}} + \kappa_{2\mathrm{D}-}$. As clearly seen in the figure, the Hermitian coupling coefficients R and I can be controlled by changing d and x.

On the other hand, the magnitude of the non-Hermitian coupling coefficient, μ, can be controlled as follows: Because μ depends on the phase θ_{DBR} of optical interference between upward-radiated light and light reflected by the DBR (see Fig. S2c), μ can be controlled by changing θ_{DBR}, where θ_{DBR} is determined by the thickness of the p-clad layer.

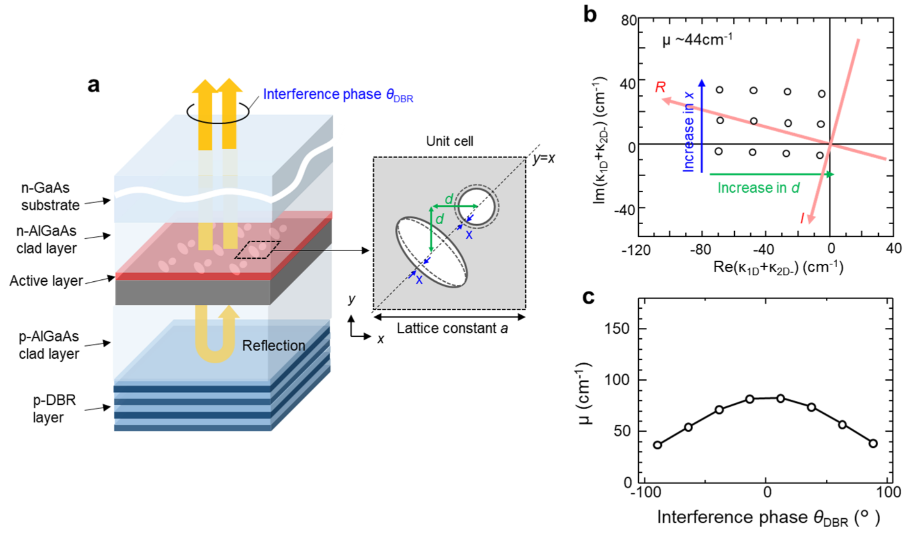

Fig. S2| Control of Hermitian and non-Hermitian coupling coefficients in a double-lattice PCSEL with a backside DBR. a, Illustration of a PCSEL with a double-lattice photonic crystal composed of an elliptical air hole and a circular air hole and a p-DBR layer. d is the lattice separation and x is a parameter for tuning the balance of the air-hole sizes. θ_{DBR} is the phase of optical interference between upward-radiated light and light reflected by the p-DBR. **b,** Calculated Hermitian coupling coefficient $\kappa_{1\mathrm{D}} + \kappa_{2\mathrm{D}-}$ (or R and I) in the complex plane, when d and x are varied. **c,** Calculated magnitude μ of the non-Hermitian coupling coefficient as a function of the interference phase θ_{DBR}.

5. Self-consistent analysis of PCSELs under CW operation

The lasing characteristics of the PCSEL under CW conditions can be simulated based on a self-consistent analysis that considers the interaction among photons, carriers, and thermal effects. Figure S3 shows the calculation flow of this analysis [S7].

First, we set the current density distribution to $J(\boldsymbol{r})$ and the initial temperature

distribution in the photonic-crystal layer to a uniform distribution $T(\boldsymbol{r})$, where $\boldsymbol{r}=(x,y)$ represents the position in the xy plane. Next, using time-dependent 3D-CWT considering carrier-photon interactions [S8], we calculate the carrier density $N(\boldsymbol{r})$ and the photon density $U(\boldsymbol{r})$ in the active layer by taking into account the temperature- and carrier-density dependences of the optical gain and the refractive index. Then, we calculate the heat density distribution $P_{\mathrm{heat}}(\boldsymbol{r})$ using the following equation:

$$P_{\mathrm{heat}}(\boldsymbol{r}) = \left(V - \frac{\hbar\omega}{e}\right) \times J(\boldsymbol{r}) + \frac{\hbar\omega d_{\mathrm{act}}}{\tau_{\mathrm{c}}} N(\boldsymbol{r}) + \frac{\hbar\omega\alpha_{\mathrm{in}} d_{\mathrm{act}}}{\Gamma_{\mathrm{act}}} \frac{c}{n_{\mathrm{g}}} U(\boldsymbol{r}), \quad \text{(S1)}$$

where V is the electrostatic potential considering the threshold voltage and the differential resistance of the PCSEL; d_{act} is the thickness of the active layer; τ_{c} is the carrier lifetime; α_{in} is the internal material loss; Γ_{act} is the confinement factor in the active layer; and n_{g} is the group index of the guided mode. The first, second, and third terms on the right-hand side of Eq. (S1) represent the heat generated by the difference between the electrostatic potential and the photon energy, by carrier recombination in the active layer, and by material absorption of the laser light, respectively.

We then change the current temperature distribution $T(\boldsymbol{r})$ according to the following equation:

$$\frac{dT(\boldsymbol{r})}{dt} = -\frac{T(\boldsymbol{r}) - T_{\mathrm{s}}(\boldsymbol{r})}{\tau_{\mathrm{T}}}, \quad \text{(S2)}$$

where τ_{T} is the time constant, which is sufficiently longer than the relaxation oscillation period, and $T_{\mathrm{s}}(\boldsymbol{r})$ is the steady-state temperature distribution expressed as:

$$T_{\mathrm{s}}(\boldsymbol{r}) = \int_{\text{heating area}} P_{\mathrm{heat}}(\boldsymbol{r}') f(\boldsymbol{r} - \boldsymbol{r}') d\boldsymbol{r}', \quad \text{(S3)}$$

where $f(\boldsymbol{r})$ is the temperature distribution of a point heat source, which is determined based

on the chip-to-heatsink packaging conditions.

Then, we update the refractive index and gain distributions in accordance with the updated temperature distribution $T(\boldsymbol{r})$. Using these updated distributions, we repeat the calculation of the carrier density $N(\boldsymbol{r})$ and optical energy density $U(\boldsymbol{r})$ until a self-consistent solution is obtained.

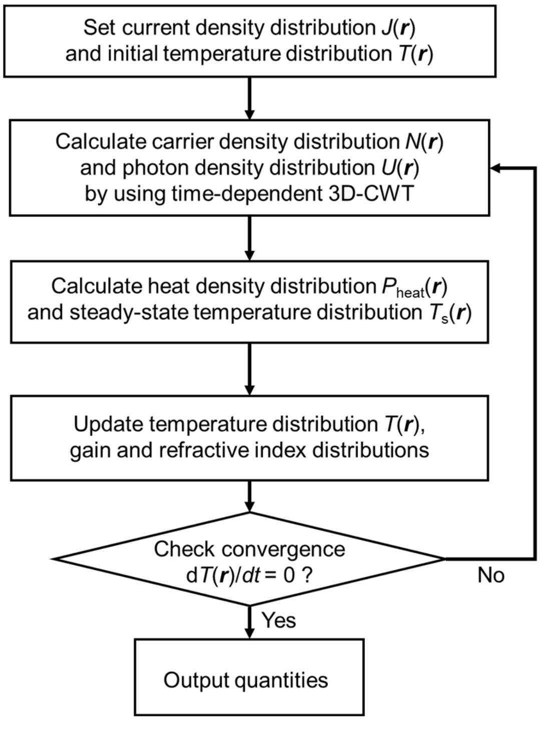

Fig. S3| Calculation flow of the self-consistent analysis of PCSELs under CW operation.

References

[S1] Wilkens, M. et al. High-efficiency broad-ridge waveguide lasers. *IEEE Photon. Technol. Lett.* **30,** 545–548 (2018).

[S2] Fiebig, C. et al. 12 W high-brightness single-frequency DBR tapered diode laser. *Electron. Lett.* **44,** 1253–1255 (2008).

[S3] Wiedenmann, D. et al. Design and analysis of single-mode oxidized VCSEL's for high-speed optical interconnects. *IEEE J. Sel. Top. Quantum Electron.* **5**, 503–511 (1999).

[S4] Contractor, R. et al. Scalable single-mode surface-emitting laser via open-Dirac singularities. *Nature* **608**, 692–698 (2022).

[S5] private communication with the authors of reference [S4].

[S6] Inoue, T. et al. General recipe to realize photonic-crystal surface-emitting lasers with 100-W-to-1-kW single-mode operation. *Nat. Commun.* **13**, 3262 (2022).

[S7] Katsuno, S. et al. Self-consistent analysis of photonic-crystal surface-emitting lasers under continuous-wave operation. *Opt. Express* **29,** 25118–25132 (2021).

[S8] Inoue, T. et al. Comprehensive analysis of photonic-crystal surface-emitting lasers via time-dependent three-dimensional coupled-wave theory. *Phys. Rev. B* **99**, 035308 (2019).
